# Protection from infection and reinfection due to the Omicron BA.1 variant in care homes

**DOI:** 10.3389/fimmu.2023.1186134

**Published:** 2023-10-23

**Authors:** Saher Choudhry, Thomas A. J. Rowland, Kamil McClelland, Erik Renz, Nalini Iyanger, J Yimmy Chow, Felicity Aiano, Shamez N. Ladhani, Anna Jeffery-Smith, Nick J. Andrews, Maria Zambon

**Affiliations:** ^1^ Virus Reference Department, UK Health Security Agency, London, United Kingdom; ^2^ London Coronavirus Response Centre, UK Health Security Agency, London, United Kingdom; ^3^ Immunisations and Countermeasures Division, UK Health Security Agency, London, United Kingdom; ^4^ Statistics, Modelling and Economics Unit, UK Health Security Agency, London, United Kingdom

**Keywords:** COVID-19, SARS-CoV-2, Omicron (BA1), care homes, outbreaks, correlate of protection, vaccine

## Abstract

**Introduction:**

Following the emergence of SARS-CoV-2 in 2020, care homes were disproportionately impacted by high mortality and morbidity of vulnerable elderly residents. Non-pharmaceutical interventions (NPIs) and improved infection control measures together with vaccination campaigns have since improved outcomes of infection. We studied the utility of past infection status, recent vaccination and anti-S antibody titres as possible correlates of protection against a newly emergent Omicron variant infection.

**Methods:**

Prospective longitudinal surveillance of nine sentinel London care homes from April 2020 onwards found that all experienced COVID-19 outbreaks due to Omicron (BA.1) during December 2021 and January 2022, despite extensive prior SARS-CoV-2 exposure and high COVID-19 vaccination rates, including booster vaccines (>70% residents, >40% staff).

**Results:**

Detailed investigation showed that 46% (133/288) of Omicron BA.1 infections were SARS-CoV-2 reinfections. Two and three COVID-19 vaccine doses were protective against Omicron infection within 2-9 weeks of vaccination, though protection waned from 10 weeks post-vaccination. Prior infection provided additional protection in vaccinated individuals, approximately halving the risk of SARS-CoV-2 infection.

**Discussion:**

Anti-S antibody titre showed a dose-dependent protective effect but did not fully account for the protection provided by vaccination or past infection, indicating that other mechanisms of protection are also involved.

## 1 Introduction

Since the start of the pandemic, United Kingdom Health Security Agency (UKHSA) (formerly Public Health England) has been monitoring a cohort of care homes as a longitudinal cohort study to understand SARS-CoV-2 exposures and transmission within this high-risk setting. By November 2021, as the Omicron (BA.1) variant emerged and spread rapidly across England, staff and residents in these care homes had already been heavily exposed to the original, alpha and delta variants that swept across the country in the previous months (1). Despite this, and high rates of three-dose vaccination (70.8% of residents and 29.4% of staff in England by 23 November 2021) (2), large outbreaks in care homes were observed in late December 2021 (3), although hospitalisation rates remained low (4).

Here, we examine the utility of three possible correlates of protection - past natural SARS-CoV-2 infection, COVID-19 vaccination, and anti-S antibody titres against three clinical outcome measures (a) infection with the Omicron variant, (b) hospitalisation and (c) infectiousness in this highly vulnerable cohort.

## 2 Methods

### 2.1 Cohort description

Since May 2020, UKHSA has conducted SARS-CoV-2 surveillance in sentinel care homes across London, England. The care homes selected for study have been previously described (5) comprising a mix of adult residential, nursing, and specialist dementia long term care facilities (LTCFs). Virologic surveillance in nine care homes, involved regular PCR and lateral flow device (LFD) testing for residents and staff in line with national guidelines, as well as periodic serology testing of staff and residents for SARS-CoV-2 spike (S) and nucleocapsid (N) antibodies (5), coupled with detailed genomic analysis of infecting virus strains. The mean age of resident at end of study = 86.3, median = 87.8, and staff average age at end of study – mean = 50.9, median = 51.1.

**Figure 1:**
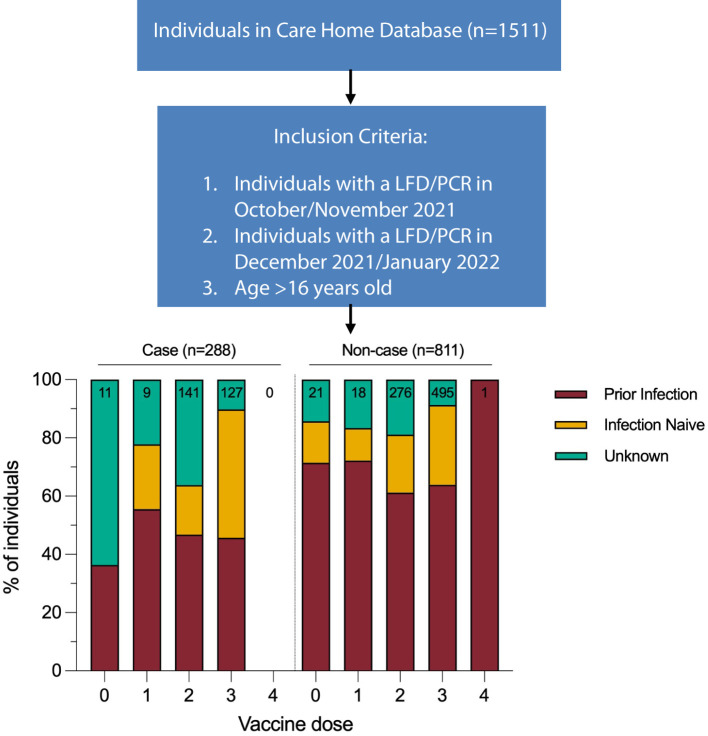
Infection status calculated for start of study period (1st December 2021). Vaccination dose for case defined as number of doses received 14 days prior to positive test result. Vaccination dose for non-cases defined as number of doses received 14 days prior to start of study period. Data for this figure was taken from **Supplementary Table 2**.

Omicron outbreaks were recorded in all nine care homes during the surveillance period. A case was defined as an individual with a positive LFD/PCR result in December 2021 or January 2022. All residents and staff with at least one PCR or LFD test in December 2021 or January 2022 (n=1,099, 78% female,) were included in the analysis of the impact of the Omicron variant. Individuals were only included if an LFD/PCR result was available from October or November 2021, to confirm that a resident or staff member was present in the care home during the surveillance period. Prior infection status was determined using PCR, LFD, and anti N and S serology results from the start of the pandemic to the start of the surveillance period (**Figure 1**).

### 2.2 Laboratory methods

Nose and throat swabs from residents and staff were sent by courier to the UKHSA Colindale Reference Laboratory for RT-PCR testing using a SARS-CoV-2 assay with E and Orf1ab gene targets as previously described (5). Some PCR testing of staff was also performed via community testing programmes in government Lighthouse laboratories. UKHSA samples with a cycle threshold (Ct) value less than 35 underwent whole genome sequencing. Viral amplicons were sequenced using Illumina library preparation kits (Nextera) and sequenced on Illumina shortread sequencing machines (Nextseq or Hiseq). The bioinformatics protocol to generate consensus sequences utilised Trimmomatic, BWA (mapping), and an in-house variant caller (quasibam) to align against a SARS-CoV-2 reference genome (NC_045512.2). Consensus sequences were generated using a depth cut-off of 20 reads. Genome lineages were allocated where the coverage of the reference genomes was 80% or more. Serological testing was performed using Roche Elecsys® Anti-SARS-CoV-2 S and N antibody testing according to manufacturers instructions.

### 2.3 Data linkage and analysis

PCR and LFD test results were extracted from the UKHSA Colindale laboratory information management system (LIMS) as well as the relevant national datasets: the Unified Sample Dataset (USD) and the Episode Level Line List. Serological data were also extracted from the UKHSA LIMS. Vaccination and demographic data, including date of death, were retrieved from the national COVID-19 vaccination database, National Immunisation Management System (NIMS). Prior infection status of an individual was allocated if there was evidence of prior infection confirmed by PCR detection or serological testing of blood samples taken prior to the start of the study. Anti N antibody detection and/or presence of anti S antibodies prior to the introduction of vaccination were taken as evidence of prior infection. Hospitalisations were identified using hospital attendance records from the national dataset [Hospital Episode Statistics, HES (6)] and NHS or Lighthouse laboratory COVID-19 test results from the UKHSA USD. Deaths were identified from the NHS Spine, which is included in the NIMS database. A&E attendances without hospitalization and elective admissions for non-COVID-19 conditions and were excluded. All hospitalisations and deaths were confirmed with the appropriate care home manager. To exclude nosocomial infections, cases were only included if any positive result was within 28 days before or after 5 days following hospital discharge, or 28 days before death. This was to ensure that the analysis for correlates of protection was focused on the impact of infection occurring in the community, rather than any nosocomial events. Data were linked together using R (v.4.2.2) in R studio (v.2022.12.0 + 353) by matching of the NHS number, names and dates of birth. Poisson regression modelling of vaccine effectiveness was undertaken in Stata (v.15) and adjusted for period (week), sex, ethnicity, care home, past infection, staff of any age/resident<70yrs/resident 70-79yrs/resident 80+yrs. Logistic regression was used to investigate the relationship between the most recent S- antibody titre taken prior to the study period and the odds of infection. Titres were grouped at <100,100-999,1000-9999,10000+. Adjustment was made for age on December 1st 2021 and week of the test, and in an exploratory analysis, past infection status was added to see whether the protection from this was mediated through S-antibodies. Ct value analysis was conducted in R using the Kruskal-Wallis test.

### 2.4 Ethics

The investigation protocol was reviewed and approved by the UKHSA Research Ethics and Governance Group (REGG) (Reference NR0204). Verbal consent for testing was obtained by care home managers from staff members and residents or their next of kin as appropriate. UK Health Security Agency (UKHSA, formerly Public Health England) has legal permission, provided by Section 3 of the Health Service (Control of Patient Information) Regulation 2002, to process patient confidential information for national surveillance of communicable diseases.

## 3 Results

**Figure 2:**
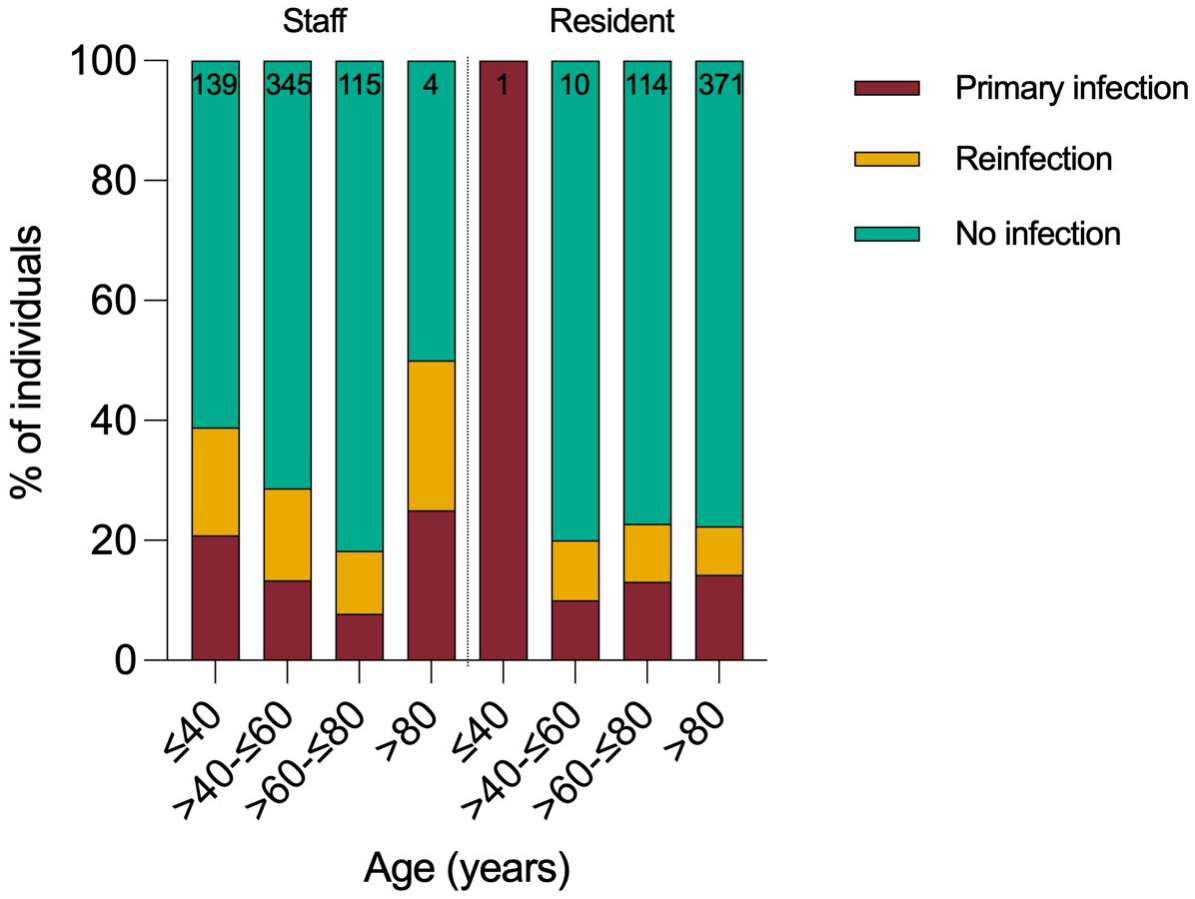
Primary Infection or reinfection during study period stratified by age and role.

In total, 112 residents and 176 staff were infected by Omicron variant during the 2-month surveillance period, giving a cumulative infection rate of 26% (288/1099). Staff had approximately twice as many PCR test results available compared to residents. 29% of staff (176/603, median age 50) and 23% of residents (85/375, median age 87) were infected (**Figure 2**). Case rates were highest in younger age-groups. Approximately half the cases (n=133, 46%) were reinfections and reinfection rates were higher in staff (52%) than residents (38%) (**Table 1**). 61% (177/288) of positive samples yielded full whole genome sequence, with 99% identified as BA.1 and no other Omicron sub-lineages identified (**Supplementary Figure 1**).

**Table 1:** Cohort infection and reinfection by age and role.

		Individuals	Cases	Case Rate (%)	Reinfection Rate (%)
**Age**	**≤40**	140	55	39	45
	**>40 - ≤60**	355	101	28	53
	**>60 - ≤80**	229	47	21	49
	**>80**	375	85	23	36
**Role**	**Staff**	603	176	29	52
	**Residents**	496	112	23	38
**Total**		**1099**	**288**	**26**	**46**

Vaccination rates were high among residents, with 17% receiving two and 77% receiving three doses compared to 52% and 43%, respectively, among staff. A small proportion of individuals were unvaccinated: 21/496 (4.2%) residents and 11/603 (1.8%) staff were unvaccinated. BNT162b2 (Comirnaty, Pfizer-BioNTech) was the most common vaccine used, representing 52%, 53%, and 90% of first, second and third doses, respectively. ChAdOx1 (Vaxzevria, AstraZeneca) made up the remainder of the primary doses, except for eight (0.7%) individuals receiving Moderna (mRNA-1273) as their primary dose and 72 (11.6%) as their third dose.

### 3.1 Protection against infection

#### 3.1.1 Protection afforded by vaccination and past infection

Analysis of relative vaccine effectiveness using Poisson regression shows that protection was significantly higher following the 3^rd^ vaccine booster compared to greater than 10 weeks after the second COVID-19 vaccine dose: 43% (95% CI, 17-61%) at 2-9 weeks after 3^rd^ vaccination and 29% (95% CI, 2-48%) at 10+ weeks post-dose 3 (**Table 2**). The second vaccine dose was also protective (49%, 95% CI, 7-73%) during the first 2-9 weeks compared to 10+ weeks post-dose 2. These findings demonstrate waning of protection against Omicron infection after both the second and third doses. Previous SARS-CoV-2 infection approximately halved the risk of reinfection, irrespective of vaccination status (**Table 2**). Regular serological assessment increased case ascertainment of symptomatic and asymptomatic infections in our cohort early during the pandemic when PCR testing was more limited. Serological testing was voluntary and only 30% of the cohort underwent serological sampling between October 2021 and February 2022. Despite this, comparison of infection rates by LFD/PCR and seropositivity/seroconversion rates in our cohort during the period indicated that <5% of infections were missed, likely because of already high immunity levels and regular PCR/LFD testing among staff and residents (7).

**Table 2:** Cohort vaccination status and vaccine effectiveness (VE) estimates.

Variable	Level	PCR positive events	Person year of follow-up	Rate per person year	Crude relative incidence vs baseline	Adjusted relative incidence vs baseline* (95% CI)	Relative vaccine effectiveness to 2 doses 10+ weeks ago (95% CI)
**Vaccination Status**	**Unvaccinated**	9	5.4	1.66	0.84	0.81 (0.40-1.64)	
	**Dose1: <2 wks ago**	2	0.2	8.42	4.24	2.73 (0.61-12.19)	
	**Dose1: 2-9 wks ago**	0	1.1	0.00	0.00	n/a	
	**Dose 1: 10+ wks ago**	7	2.7	2.57	1.29	0.95 (0.42-2.12)	
	**Dose2: <2 wks ago**	0	0.7	0.00	0.00	n/a	
	**Dose2: 2-9 wks ago**	12	8.8	1.37	0.69	0.51 (0.27-0.93)	49% (7-73)
	**Dose2: 10+ wks ago**	101	50.9	1.99	baseline	baseline	baseline
	**Dose3: <2 wks ago**	13	7.7	1.68	0.85	0.79 (0.44-1.41)	
	**Dose3: 2-9 wks ago**	41	56.3	0.73	0.37	0.57 (0.39-0.83)	43% (17-61)
	**Dose 3: 10+ wks ago**	103	87.7	1.17	0.59	0.71 (0.52-0.98)	29% (2-48)
**Past Infection**	**No**	82	55.6	1.47	baseline	baseline	
	**Yes**	133	133.9	0.99	0.67	0.48 (0.36-0.65)	
	**Unknown**	73	32.0	2.28	1.54	1.11 (0.78-1.56)	

*Model included period (week), sex, ethnicity, care home, past infection, staff of any age/resident<70yrs/resident 70-79yrs/resident 80+yrs.

#### 3.1.2 Protection afforded by anti-S antibodies

**Figure 3:**
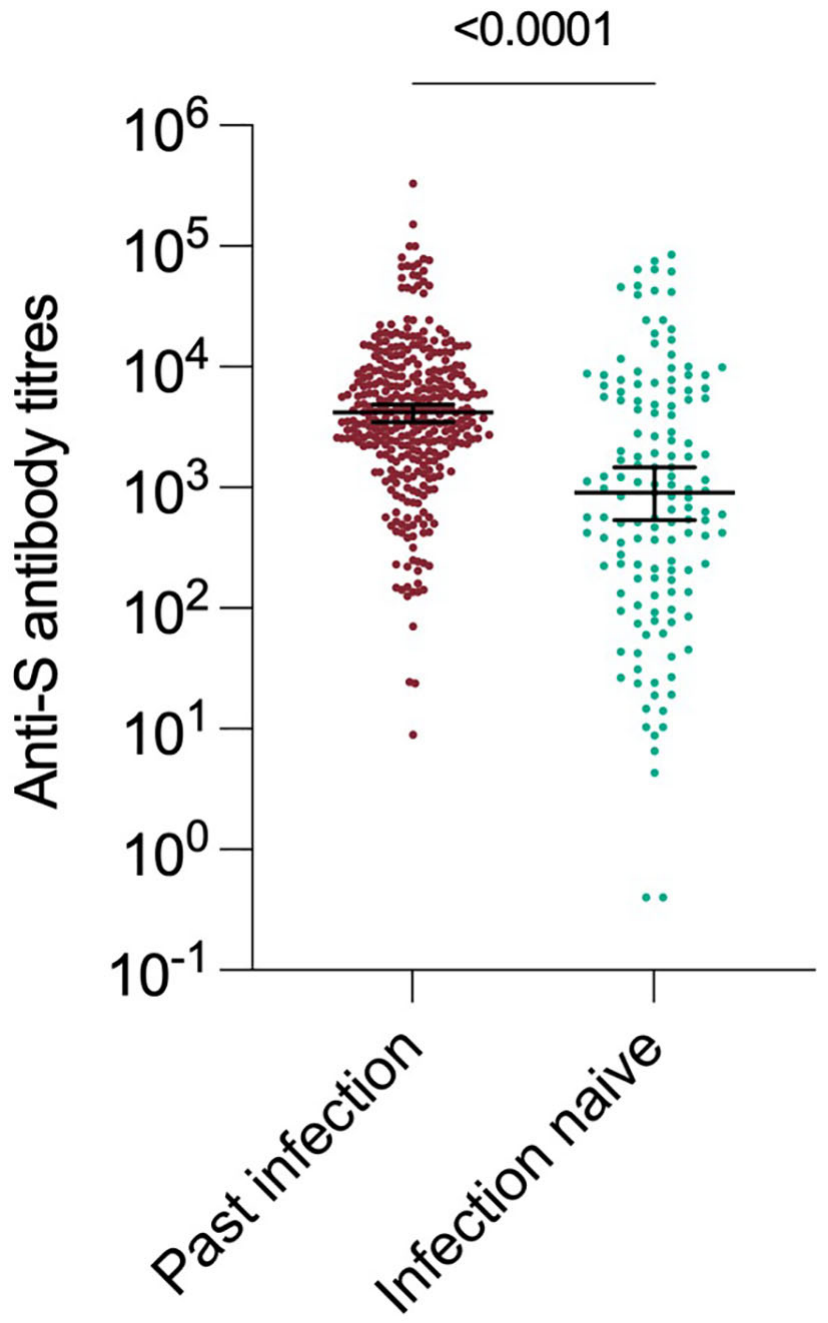
Distribution of Antibody titres.

Anti-S antibody titres from serum collected in the 90 day period(1 Sept – 1 Dec 2022) immediately before the Omicron variant emerged were a strong predictor of past infection regardless of vaccination status. Those individuals who had previously been infected with an earlier variant had significantly higher anti S antibody titres (median 4170 vs 910, p < 0.0001) (**Figure 3**). The assessment of recent S-antibody on the odds of infection showed reductions in the odds of infection of 49%, 74% and 69% for those with titres of 100-999, 1000-9999, 10000+ compared to titres of <100 respectively (odds ratios 0.51 95% CI: (0.21-1.19), 0.26 (012-0.59), 0.31(0.12-0.76)). This indicates evidence of increased protection up to titres above 1000 and then a plateau, based on samples taken in the month prior to the study period. These antibodies will have been generated by vaccination and/or infection but likely do not mediate all the protective effects. If prior infection status is included in the model this still shows evidence of protection on subsequent infection (odds ratio 0.60, 95% CI (0.35-1.01)). It was not possible to add vaccination status to the model due to its high level of association with S-antibody titres.

### 3.2 Protection against severe disease`

Clinical outcome data is provided in **Table 3**. All hospitalizations and fatalities occurred in residents. Hospitalisation rates were much higher among unvaccinated individuals than those who had received two or more vaccine doses (37% vs 3%), as was the case fatality rate (36% vs 5%). Exploration of the relationship between antibody titres, past infection and severe disease was limited by the small numbers of individuals with severe disease.

**Table 3:** Clinical outcomes within cohort by role, age, number of vaccine doses and past infection status.

		Hospitalised	Died	Hospitalisation Rate (%)	Case Fatality Rate (%)
**Role**	**Staff (n=176)**	0	0	0.0	0.0
	**Resident (n=112)**	12	18	10.7	16.1
**Age**	**60 – 80 (n=47)**	3	3	6.4	6.4
	**>80 (n=85)**	9	15	10.6	17.6
**Vaccination Status**	**Unvaccinated (n=11)**	3	4	27.3	36.4
	**1 Dose (n=9)**	0	1	0.0	11.1
	**2 Doses (n=141)**	2	5	1.4	3.5
	**3 Doses (n=127)**	7	8	5.5	6.3
**Past Infection**	**No (n=82)**	2	4	2.4	4.9
	**Yes (n=133)**	4	4	3.0	3.0
**Total (n=288)**		**12**	**18**	**4.2**	**6.3**

### 3.3 Protection against infectiousness

Cycle threshold (Ct) value obtained during RT PCR testing from samples taken early in infection gives an indication of the magnitude of viral shedding. Several studies have used this as a proxy for infectiousness during primary infection (8). In this study, Ct values were significantly lower (indicating higher viral load) in infection-naive compared with previously infected individuals, (median 24.9 vs 28.4, p = 0.004) and did not correlate with role (staff vs resident) (**Supplementary Figure 2**). In all cases who underwent PCR testing, no significant difference in Ct value distributions was noted by vaccination status (two versus three doses). There were insufficient samples to compare Ct values among unvaccinated cases or those who had only received one dose.

## 4 Discussion

Whilst Omicron’s increased transmissibility with high infection and reinfection rates in the community is well-described (9), less is documented about transmission of this variant in high-risk, highly vaccinated settings. While reducing the force of infection, high rates of prior infection and vaccination did not prevent considerable transmission occurring in all sentinel care homes, despite longstanding implementation of stringent infection control measures and social distancing in this setting. The 46% reinfection rate with Omicron was substantially higher compared to the 3% for the Alpha variant in the same cohort in January 2021 (5), a likely consequence of antibody waning over time since primary infection, combined with immune evasion by the Omicron variant.

Modelled estimates of reinfection with Omicron in the community are similar to the rates that we observed, higher than the estimate of 9.5% derived from national operational testing data (4, 10, 11). Reinfection rates were higher in staff versus residents, likely explained by lower third booster vaccination rates, higher exposure risk in the community likely arising as a result of increased connectivity and increased testing improving ascertainment. Of the 133 reinfections, 67% had primary infection in the pre-Alpha period and 17% in the Alpha and Delta periods, respectively. Notably, 6/133 reinfections were third infections, first with pre-Alpha or Alpha, followed by Delta and then Omicron.

Three possible indicators of protection showed varying levels of correlation against selected clinical outcome measures. Our finding of higher protection with increasing doses of COVID-19 vaccines is consistent with other reports of protection against Omicron infection through vaccination (10) and reassuring about the value of booster vaccine doses. Given the high rates of prior infection in our cohort, our finding that the combination of prior infection and vaccination may provide improved protection against Omicron infection than vaccination alone is also noteworthy. Outside the care home setting, prior infection has been estimated to provide 60% protection against reinfection with Omicron compared to Alpha, Beta or Delta variants (12, 13), with vaccination providing additional protection in previously-infected individuals (11).

We did not find a correlation between peak viral shedding (for which Ct value is a proxy) and vaccination status. This is consistent with other studies that have shown vaccination reduces the duration and overall amount of viral shedding rather than the peak of viral shedding (14, 15). Our finding of higher Ct values suggestive of lower viral shedding in previously infected individuals is consistent with findings in other community studies outside the care home setting (16). The duration of infectiousness does not reliably correlate with the magnitude of the peak of viral shedding (15, 17).

In this study, we have measured anti-S antibodies as a proxy for neutralizing antibody (**Figure 3**). Anti-S antibody titre was a dose-dependent correlate of protection from infection. This is consistent with observational studies during the first waves of the pandemic, where levels of neutralising antibody titres were suggested as possible correlate of protection (18, 19). Prior work in this cohort has also shown antibody titres to be correlated with protection against antigenically similar variants (5). The S protein of Omicron variant has a high number of changes and demonstrates significant antigenic distance from earlier variants (20).This work shows that high antibody titres remain protective even in the context of exposure to an antigenically-distant variant.

However, antibody titre was not sufficient to fully explain the protective effect of vaccination or past natural infection and therefore we hypothesise some protective effects must be exerted through pre-existing adaptive or innate immunity mechanisms, including cellular response. Unmeasured contributors to protection may also include innate control mechanisms and mucosal antibodies These require further study. The relative contribution between different classes of strain specific antibodies to viral S protein Receptor Binding Domain (RBD) or N terminal domain vs cross reactive neutralizing antibodies requires detailed analysis of antibody repertoire following primary and secondary infection and the impact of vaccination (21). Examination of the profile of antibody repertoire following boosting by vaccination or re-infection should yield further insights into the nature of antibody correlates of protection. The role or contribution of mucosal antibody in protection from infection has not been considered in this study.

The very low overall hospitalisation rate (<5%) in our cohort during large Omicron outbreaks represents a significant improvement from the pre-vaccine period (1). Emerging data continues to demonstrate decoupling between viral infection or reinfection and severity of disease as measured by hospitalisation during the Omicron and related variants wave. Our study confirming this finding in high-risk settings is reassuring and consistent with others (1, 22) and highlights the importance of ensuring high vaccine uptake and repeated regular boosters to ensure continued protection in this vulnerable cohort (23). Establishing the intrinsic severity of future newly emerging variants will increasingly rely upon animal or *in vitro* models as a result of high degree of population exposure, residual immunity and impact of vaccination.

### 4.1 Strengths and limitations

Our assessment of Omicron infection risk by prior infection status, vaccination status and time since vaccination was only possible because of the longitudinal nature of our study since the start of the pandemic. There are, however, some limitations. The data included here was censored on 31 January. Therefore, while this paper includes the most intense outbreak period and the majority (>90% of the cases), outbreaks continued at a low level into March 2022. Some outbreaks had not completely terminated at the end of the study period, likely underestimating by a small margin the overall case and reinfection rates.

The higher rate of PCR testing of staff could potentially lead to improved ascertainment of infection and identification of re-infection. Overall, the rates of serological testing were similar between staff and residents, and therefore any bias towards improved detection in staff would be based on the probability of detecting infection by more frequent PCR testing, rather than assessment of past infection status due to serological investigations. This higher ascertainment in staff will not bias estimates of vaccine effectiveness or the effect of past infection since it will apply equally irrespective of vaccination status or past infection status and adjustment is made in the analysis for being staff/resident.

Hospitalisations and deaths were attributed to COVID-19 by temporal correlation, and therefore we do not distinguish ‘deaths with COVID-19’ from ‘deaths from COVID-19’. This may overestimate the number of severe outcomes from COVID-19 infection experienced by the cohort. We were only able to assess protection against the Omicron BA.1 subvariant and, therefore, our findings are not considered for the subsequently dominant BA.4/BA.5 subvariants.

## Data availability statement

Genomic data is available from GISAID. The other datasets presented in this article are not readily available because they contain person identifiable information collected and collated as part of an outbreak response under Section 3 of the UK Health Service (Control of Patient Information) Regulations 2002. Requests to access the datasets should be directed to the corresponding author.

## Ethics statement

The studies involving humans were approved by PHE Research and Ethics Governance Committee. The studies were conducted in accordance with the local legislation and institutional requirements. Written informed consent for participation in this study was provided by the participants’ legal guardians/next of kin.

## Author contributions

NI, JC, SL AJ-S and MZ conceived and designed the study. SC also contributed to study design. TR, KM, ER and FA were responsible for data acquisition. SC, TR, KM, NA and MZ conducted data analysis and interpretation, ER, FA and AJS also contributed to data analysis. SC, TR, MZ and KM wrote the manuscript. All authors critically revised the manuscript. All authors contributed to the article and approved the submitted version.

## Supplementary material

Supplementary Figure 1 Genomic analysis of an Omicron outbreak within a single care home indicating several different introductions, and transmission of the same variant between staff and residents with varying degrees of vaccination.
