# Supplementary material for: Protection from infection and reinfection due to the Omicron BA.1 variant in care homes
Source: Front Immunol. 2023 Oct 23;14:1186134. doi: 10.3389/fimmu.2023.1186134 (PMC10627010; doi:10.3389/fimmu.2023.1186134)
Supplement: Supplementary file 1 [file Image_2.pdf]

Supplementary Figure 2

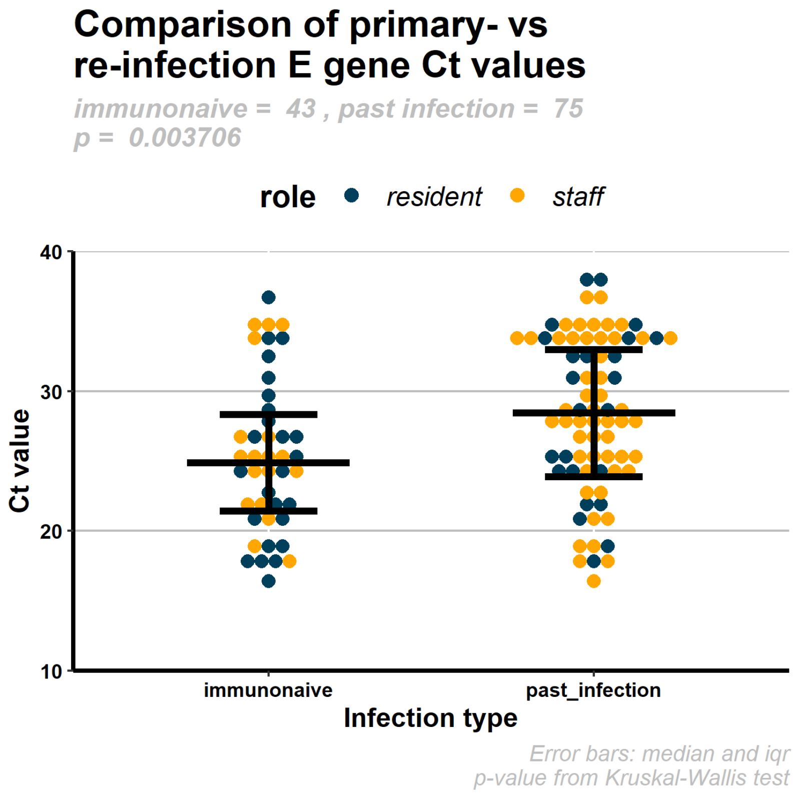

Cohort case samples tested by PCR were used for this analysis.

Nasopharyngeal samples were collected into Virus Transport media (VTM). Following automated extraction of viral RNA from the sample, realtime qPCR was perfomed using two sets of primers and probes to detect SARS-CoV-2 in the ORF1Ab assay target , and Sarbecoviruses including SARS CoV-2 in the E gene target. ABI QuantStudio 7 Flex instrument was the platform, using the Invitrogen TaqPath Multiplex Master Mix. Cycle threshold (Ct) values were determined using QuantStudio software, with the threshold set individually for each channel according to the exponential growth curves and above background fluorescence of negative controls. All assay results were analysed using pre-determined threshold values and SARS-CoV-2 was reported as detected if either ORF1ab or E gene is detected at Ct < 35, or if both targets are detected at Ct >35.
