# Supplementary material for: Protection from infection and reinfection due to the Omicron BA.1 variant in care homes
Source: Front Immunol. 2023 Oct 23;14:1186134. doi: 10.3389/fimmu.2023.1186134 (PMC10627010; doi:10.3389/fimmu.2023.1186134)
Supplement: Supplementary file 3 [file Table_2.pdf]

Supplementary Table 2

| N=1099 | Vaccination Dose+ | Infection Status* | | |
|---|---|---|---|---|
| | | Prior Infection | Infection Naïve | Unknown |
| Case (n=288) | 0 | 4 | 0 | 7 |
| | 1 | 5 | 2 | 2 |
| | 2 | 66 | 24 | 51 |
| | 3 | 58 | 56 | 13 |
| | 4 | 0 | 0 | 0 |
| Non-Case (n=811) | 0 | 15 | 3 | 3 |
| | 1 | 13 | 2 | 3 |
| | 2 | 169 | 55 | 52 |
| | 3 | 316 | 136 | 43 |
| | 4 | 1 | 0 | 0 |

*Prior infection status was calculated for the start of study period (1st December 2021).
+Vaccination dose status for cases defined as number of doses received 14 days prior to positive test result. Vaccination dose status for non-cases defined as number of doses received 14 days prior to start of study period. Number of Individuals included in the study (N=1099; Males =241)
