# Supplementary material for: Protection from infection and reinfection due to the Omicron BA.1 variant in care homes
Source: Front Immunol. 2023 Oct 23;14:1186134. doi: 10.3389/fimmu.2023.1186134 (PMC10627010; doi:10.3389/fimmu.2023.1186134)
Supplement: Supplementary Figure 1 — Genomic analysis of an Omicron outbreak within a single care home indicating several different introductions, and transmission of the same variant between staff and residents with varying degrees of vaccination. [file Image_1.pdf]

Supplementary Figure 1

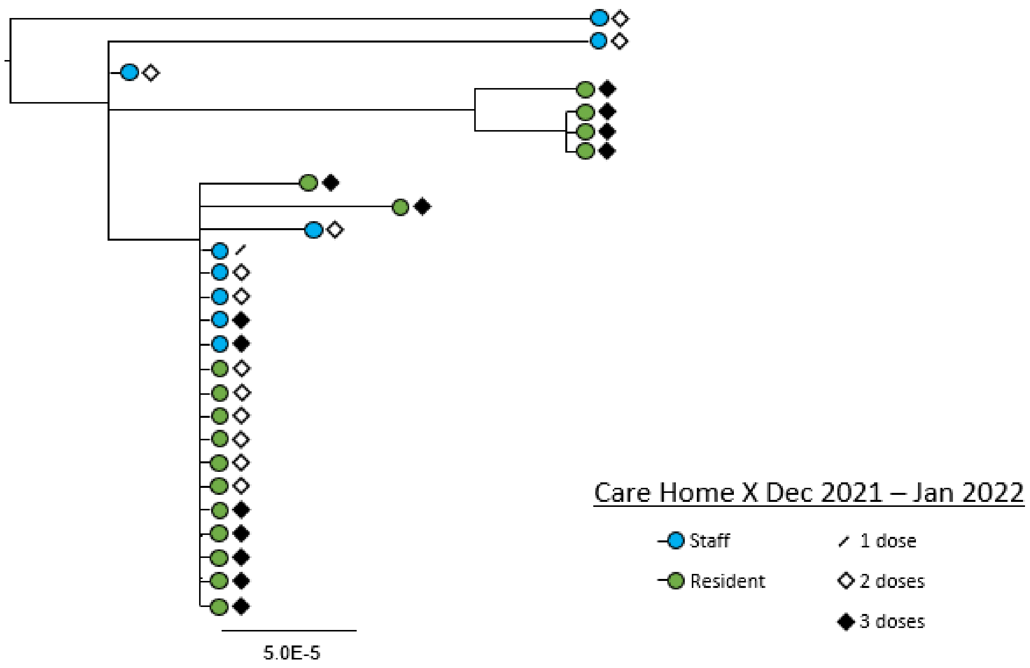

Genomic analysis of an Omicron outbreak within a single care home indicating several different introductions, and transmission of the same variant between staff and residents with varying degrees of vaccination. Viral amplicons were sequenced using Illumina library preparation kits (Nextera kit, Illumina, Cambridge, UK) and sequenced on Illumina short-read sequencing machines (Hiseq, Illumina, Cambridge, UK). The bioinformatics protocol to generate consensus sequences utilised Trimmomatic, BWA (mapping), and an in-house variant caller (quasibam) to align against a SARSCoV-2 reference genome. Consensus sequences were generated using a depth cut-off of 20 reads and maximum likelihood phylogenetic trees derived using IQtree (version 2.04). Genomes were included in analysis where the coverage of the reference genomes was ≥80%. Completed viral genomes were deposited in GISAID (Supplementary Table 1).
